# Simulation of the Pyrolysis Process of Cyclohexane-Containing Semi-Aromatic Polyamide Based on ReaxFF-MD

**DOI:** 10.3390/polym17121593

**Published:** 2025-06-06

**Authors:** Xiaotong Zhang, Yuanbo Zheng, Qian Zhang, Kai Wu, Qinwei Yu, Jianming Yang

**Affiliations:** 1 Xi’an Modern Chemistry Research Institute, Xi’an 710065, China; zhangxt204@163.com (X.Z.); zyb13102235565@163.com (Y.Z.); qz450945428@163.com (Q.Z.); tjuk_wu@outlook.com (K.W.); qinweiyu204@163.com (Q.Y.); 2 State Key Laboratory of Fluorine & Nitrogen Chemicals, Xi’an 710065, China

**Keywords:** ReaxFF-MD, semi-aromatic polyamide, cyclohexane, pyrolysis

## Abstract

Cyclohexane-containing semi-aromatic polyamides (c-SaPA) exhibit excellent comprehensive properties. Existing studies predominantly focus on synthesis and modification, while fundamental investigations into pyrolysis mechanisms remain limited, which restricts the development of advanced materials for high-performance applications such as automotive and energy systems. This study employs Reactive Force Field Molecular Dynamics (ReaxFF-MD) simulations to establish a pyrolysis model for poly(terephthaloyl-hexahydro-m-xylylenediamine) (PHXDT), systematically probing its pyrolysis kinetics and evolutionary pathways under elevated temperatures. The simulation results reveal an activation energy of 107.55 kJ/mol and a pre-exponential factor of 9.64 × 10^13^ s^−1^ for the pyrolysis process. The primary decomposition pathway involves three distinct stages. The first is initial backbone scission generating macromolecular fragments, followed by secondary fragmentation that preferentially occurs at short-chain hydrocarbon formation sites alongside radical recombination. Ultimately, the process progresses to deep dehydrogenation, carbonization, and heteroatom elimination through sequential reaction steps. Mechanistic analysis identifies multi-pathway pyrolysis involving carboxyl/amide bond cleavage and radical-mediated transformations (N-C-O, C-C-O, OH· and H·), yielding primary products including H_2_, CO, H_2_O, CH_3_N, C_2_H_2_, and C_2_H_4_. Crucially, the cyclohexane structure demonstrates preferential participation in dehydrogenation and hydrogen transfer reactions due to its conformational dynamic instability and low bond dissociation energy, significantly accelerating the rapid generation of small molecules like H_2_.

## 1. Introduction

Semi-aromatic polyamides (SaPA) represent a class of polyamides incorporating both rigid aromatic units and flexible methylene segments within their molecular backbone [1]. Combining the advantageous characteristics of aliphatic polyamides (e.g., PA66) and fully aromatic polyamides (e.g., PPTA), SaPA achieves optimal balance among thermal resistance, mechanical strength, and processability [2,3,4,5]. Cyclohexane-containing semi-aromatic polyamides (c-SaPA) exhibit enhanced performance attributes due to the cyclohexane moieties, which impart superior structural rigidity compared to linear aliphatic chains. The cyclohexane moieties also introduce cis-trans isomerization effects that disrupt polymer chain regularity [6,7]. These characteristics confer exceptional thermal stability, optical transparency, weather resistance, and gas barrier properties to c-SaPA, rendering it a promising candidate for advanced applications such as photovoltaic encapsulation and automotive lightweighting [8,9,10,11,12,13,14,15,16,17].

Current research primarily focuses on synthesizing c-SaPA and developing modification strategies through functional group incorporation (e.g., trifluoromethyl, bicyclohexyl, and caprolactam units), which significantly enhance thermal properties, mechanical performance, and processability [18,19,20,21,22]. However, in practical applications such as photovoltaic panel encapsulation and automotive engine compartments, c-SaPA is subjected to combined environmental stressors. These include prolonged ultraviolet irradiation and thermo-oxidative aging, which induce structural degradation [23,24,25]. While material engineering advancements have enhanced intrinsic properties, comprehensive mechanistic understanding of failure modes under operational extremes remains incomplete. Establishing theoretical models for c-SaPA pyrolysis processes is therefore critical to bridge these limited theoretical foundations and provide molecular dynamics (MD) insights for stability-oriented material design.

Conventional characterization techniques (e.g., TG-IR-GC/MS) can identify pyrolysis products like benzene derivatives but fail to track MD features at femtosecond timescales, including radical migration dynamics and transient evolution of metastable intermediates [26,27,28,29,30]. Breakthroughs in pyrolysis mechanism studies have been revolutionized by advances in Reactive Force Field Molecular Dynamics (ReaxFF-MD) simulations [31,32,33]. By employing bond order-distance coupled potential functions, ReaxFF-MD overcomes the inherent limitations of classical force fields in describing bond cleavage/reformation, enabling full-cycle dynamic tracking of bond rupture sequences, radical chain reactions, and product formation networks [34,35,36]. Compared to conventional MD methods, ReaxFF-MD reduces quantum chemical computation complexity by 2–3 orders of magnitude while maintaining sub-picosecond temporal resolution, facilitating simulations of macromolecular systems [37,38,39,40].

ReaxFF-MD has successfully elucidated critical mechanisms: identifying pyrolysis sites in meta-aramid fibers (aromatic-N and C=O bond cleavage), revealing co-pyrolysis synergies in automotive polypropylene blends (EPDM-enhanced tar formation, POE-promoted C_2_H_2_/C_3_H_4_ yields), optimizing bio-based polyester elastomer stability (C-O bond-dominated degradation), and deciphering atomic oxygen resistance mechanisms in polyimide/POSS composites (CF_3_-SiO_2_ synergistic effects) [41,42,43,44]. Notably, this technique has quantified femtosecond-scale β-scission dynamics in polyethylene and activation entropy variations during carbonate group rearrangements in polycarbonates, laying theoretical foundations for universal polymer pyrolysis models [45,46]. Nevertheless, the role of cyclohexane units in c-SaPA pyrolysis remains unclarified, constituting a critical scientific barrier to accurate thermal behavior prediction.

This study aims to elucidate the pyrolysis process of c-SaPA through ReaxFF-MD simulations. By constructing a molecular model of poly(terephthaloyl-hexahydro-m-xylylenediamine) (PHXDT, a representative c-SaPA compound), we systematically simulated its pyrolysis processes across varying temperatures, performed pyrolysis kinetic calculations, and analyzed the distribution patterns and formation pathways of primary decomposition products. The findings elucidate the influence of cyclohexane structure on the pyrolysis behavior of PHXDT. These MD-based theoretical insights support the design of stable c-SaPA materials for extreme environments.

## 2. Computational Methods and Simulation Details

**Figure 1:**
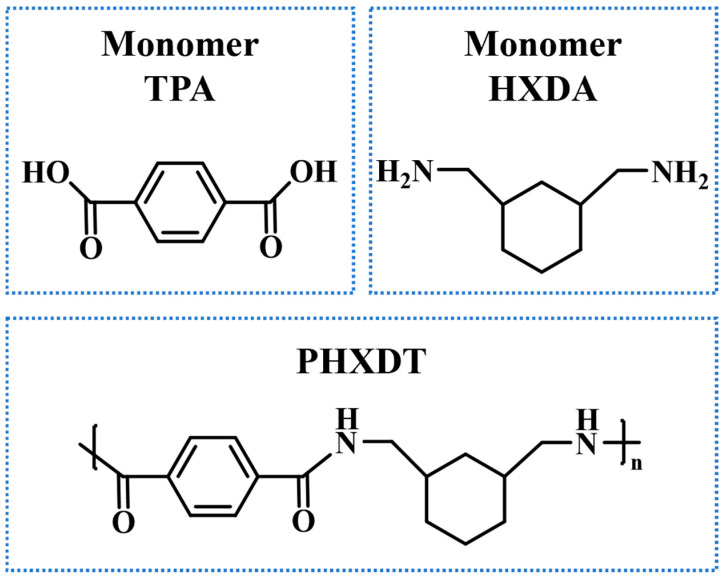
Monomer and structural formula of PHXDT.

This study focuses on c-SaPA, specifically the amorphous structure PHXDT, with the chemical formula (C_16_H_20_N_2_O_2_)_n_ [47]. The repeating unit comprises 16 carbon atoms, 20 hydrogen atoms, 2 nitrogen atoms, and 2 oxygen atoms. Figure 1 depicts the molecular structures of the two monomers, terephthalic acid (TPA) and hexahydro-m-xylylenediamine (HXDA), along with the complete structural formula of the resulting polyamide PHXDT. The introduced cyclohexane moiety synergizes with aromatic rings to form a heterocyclic-aromatic coupled architecture, which modulates the material’s thermal stability and pyrolysis kinetics, providing a structural foundation for precisely modeling the mechanistic role of cyclic structures in pyrolysis processes.

**Figure 2:**
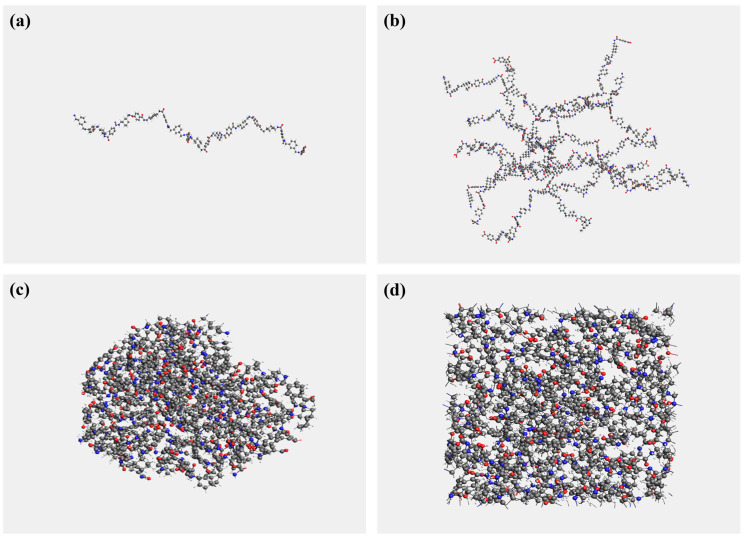
(**a**) Single-chain PHXDT model, (**b**) multi-chain PHXDT model, (**c**) NPT-optimized PHXDT model, (**d**) NVT-optimized PHXDT model.

A molecular model balancing computational accuracy and cost-effectiveness is constructed through a two-stage approach. First, a linear chain containing 10 repeating units (*n* = 10) was geometrically optimized to obtain the lowest-energy conformation [48,49]. Subsequently, a system comprising eight PHXDT chains was constructed via MD simulations. This multi-chain model not only satisfies periodic boundary conditions but also effectively replicates the statistical mechanical behavior of polymer chains in real materials. The single-chain and multi-chain PHXDT models are depicted in Figure 2a,b,with black spheres representing carbon atoms, red corresponding to oxygen atoms, white denoting hydrogen atoms, and blue indicating nitrogen atoms. The pyrolysis process of PHXDT was simulated using the Amsterdam Modeling Suite (AMS). Prior to pyrolysis simulations, the constructed PHXDT models underwent energy minimization via geometry and energy optimization in the ADF module to ensure a stable, low-energy initial configuration.

The thermal pyrolysis simulation was conducted using the C/H/O/N-2019 ReaxFF force field parameter set developed by Kowalik and co-authors, which was specifically optimized for polymer systems containing carbon, hydrogen, oxygen, and nitrogen [37]. This force field has been successfully applied in thermodynamic process simulations including polyimide carbonization and epoxy resin gasification [50,51]. Additionally, systematic monitoring of energy conservation and dynamic stability throughout the equilibration phase ensured physically consistent simulation trajectories, with no non-physical artifacts observed in the system’s evolution. The simulation protocol consisted of three stages: First, NPT equilibration was performed at 300 K and 0.1 MPa to obtain the structurally optimized PHXDT molecular chain model (Figure 2c) [52]. Subsequently, NVT equilibration was implemented at 300 K to ensure the system reached full thermodynamic equilibrium (Figure 2d) [53]. Finally, pyrolysis simulations were conducted at five temperatures (2000 K, 2500 K, 3000 K, 3500 K, and 4000 K) for 100 ps to systematically track the dynamic decomposition process of PHXDT.

**Figure 3:**
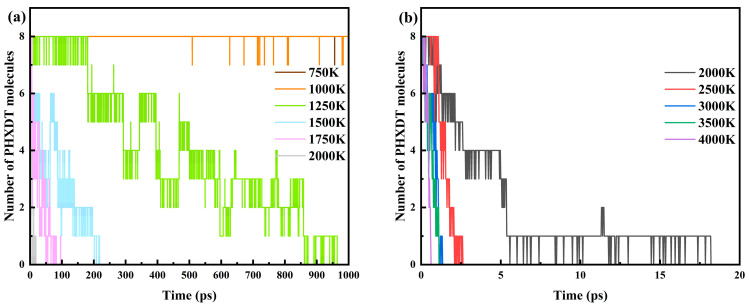
Evolution of molecular number of PHXDT at different pyrolysis temperatures. (**a**) 750–2000 K, (**b**) 2000–4000 K.

It should be noted that the rationality of employing elevated temperatures (1500–4000 K) in ReaxFF-MD simulations has been fully validated by numerous studies to accelerate reaction rates and reduce computational time scales, while maintaining consistency between key reaction pathways under high-temperature conditions and low-temperature experimental results [48]. The simulated product distributions have been demonstrated to show good agreement with experimental characterization data [54,55]. Preliminary simulations conducted across two temperature ranges (750–2000 K and 2000–4000 K, Figure 3) revealed distinct pyrolysis behaviors. In the 750–1000 K range, PHXDT exhibited no significant pyrolysis trends within 1000 ps, with complete decomposition times exceeding the scope of conventional MD simulations, rendering insufficient data for pyrolysis process analysis. While decomposition was achievable within 1000 ps in the 1250–2000 K range, comprehensive pyrolysis product information remained elusive. In contrast, under 2000–4000 K conditions, pyrolysis completed within 20 ps, enabling robust analysis of pyrolysis products and mechanisms, thereby validating the theoretical framework of the methodology employed in this study. Finally, pyrolysis kinetic parameters were calculated, and the distribution patterns and evolution pathways of primary pyrolysis products were determined using visualization modules.

## 3. Results and Discussion

### 3.1. Calculation of PHXDT Pyrolysis Kinetics

The molecular population of PHXDT exhibited oscillatory behavior during pyrolysis, driven by bond recombination processes mediated through diffusion dynamics. As illustrated in Figure 3a, the total decomposition times of PHXDT at 1250, 1500, 1750, and 2000 K were approximately 948.325, 218.450, 82.500, and 18.200 ps, respectively. In Figure 3b, the decomposition times at 2000, 2500, 3000, 3500, and 4000 K were measured as approximately 18.200, 2.625, 1.350, 1.125, and 0.625 ps, respectively. Elevated temperatures significantly enhance molecular kinetic and potential energies, thereby accelerating decomposition rates, shortening reaction times, and driving a rapid decline in PHXDT population.

Subsequently, the thermal decomposition kinetics of PHXDT are evaluated. The first-order kinetics of pyrolysis, investigated through the consumption rate of the reactant, have been thoroughly reported in extensive studies [41,46]. The consumption rate of PHXDT during the initial decomposition stage is proportional to its instantaneous concentration, and the kinetic model employed in such studies assumes complete conversion of reactants to products. Consequently, the concentration of PHXDT is directly characterized by the number of molecules (N_t_) within the system, with the initial molecular count (N_0_) set as 8. The apparent rate constant (k) under each isothermal condition is calculated by linear regression fitting of ln(N_t_/N_0_) versus decomposition time (t) (Equation (1)). To further derive activation parameters, the Arrhenius equation (Equation (2)) is applied by fitting ln k versus the reciprocal of the thermodynamic temperature (1/T), from which the activation energy (E_a_) and pre-exponential factor (A) are determined. It is specifically emphasized that the k values at each temperature point are calculated by averaging multiple independent simulations to minimize the influence of random errors on the Arrhenius fitting.

lnN_0_ − lnN_t_ = kt, (1)

ln k = ln A − E_a_/RT, (2)

**Figure 4:**
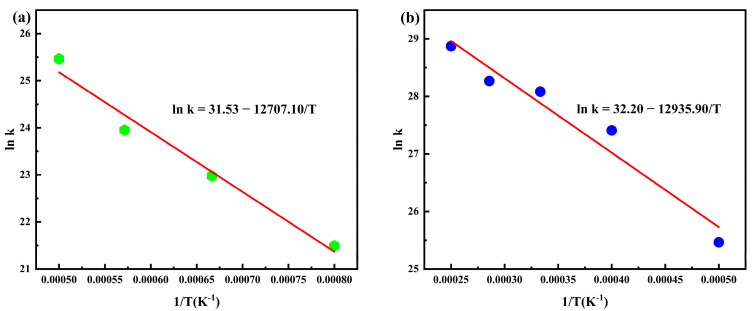
Arrhenius model of PHXDT at different pyrolysis temperatures: (**a**) 1250–2000 K, (**b**) 2000–4000 K.

As shown in Figure 4a, the fitted slope and intercept under 750–2000 K conditions were −12,707.10 K·s^−1^ and 31.53 s^−1^, respectively, yielding an activation energy (E_a_) of 105.65 kJ/mol and a pre-exponential factor (A) of 4.94 × 10^13^ s^−1^. For the 2000–4000 K range in Figure 4b, the fitted slope and intercept were −12,935.9 K·s^−1^ and 32.20 s^−1^, corresponding to E_a_ = 107.55 kJ/mol and A = 9.64 × 10^13^ s^−1^. The activation energies showed fundamental consistency between the two temperature ranges, with pre-exponential factors maintaining agreement in order of magnitude, which aligns with Arrhenius behavior.

Compared to the meta-aramid pyrolysis parameters reported by Yin and co-researchers (E_a_ = 121.45 kJ/mol, A = 6.59 × 10^13^ s^−1^), the parameters obtained under 2000–4000 K conditions exhibited an activation energy difference of approximately 11%, while the pre-exponential factors remained consistent in order of magnitude [41]. This parameter discrepancy might be attributed to the molecular structural characteristics of the two polyamides: the presence of specific substituents (e.g., cyclohexane structures) in PHXDT molecular chains was considered to potentially reduce the pyrolysis energy barrier, whereas the rigid aromatic stacking structures in meta-aramid were suggested to require higher activation energy for dissociation initiation. Despite numerical differences, both systems were found to follow the typical pyrolysis behavior of aromatic polymers characterized by high activation energies and high pre-exponential factors, simultaneously validating the reliability of the simulation methodology.

### 3.2. Analysis of the PHXDT Pyrolysis Reaction Process

**Figure 5:**
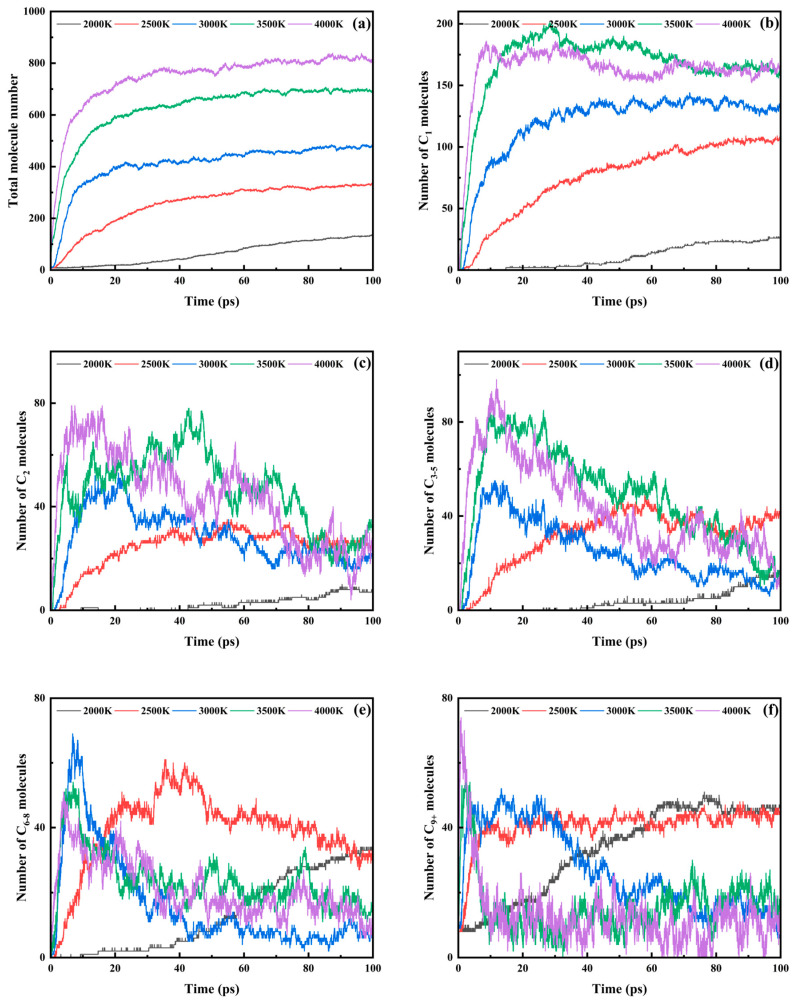
Number of molecules at different pyrolysis temperatures for (**a**) total fragments in the system, (**b**) C_1_, (**c**) C_2_, (**d**) C_3–5_, (**e**) C_6–8_, and (**f**) C_9+_.

To investigate the pyrolysis mechanisms of PHXDT, ReaxFF-MD simulations were performed across five temperature gradients (2000, 2500, 3000, 3500, and 4000 K) over a 100 ps duration. All product species, along with the molecular quantities of carbon-containing products, were statistically analyzed. The quantitative analysis results are presented in Table 1. Specifically, Figure 5a illustrates the temporal evolution of total molecular counts, revealing a pronounced temperature-dependent regulation of product distribution. As temperature increased from 2000 K to 4000 K, the onset time for molecular population growth decreased sharply from 1.05 ps to 0.025 ps, while the time required to reach terminal molecular counts at 2000 K (100 ps) was reduced to 0.425 ps at 4000 K. Concurrently, the final molecular population exhibited exponential growth from 135 to 816, consistent with Arrhenius kinetics. Elevated thermal energy significantly accelerated backbone dissociation and promoted the formation of small-molecule products (e.g., CO and H_2_), driving rapid molecular proliferation.

To systematically analyze pyrolysis pathways, carbon-containing products were categorized based on atomicity: C_1–2_ (small molecular fragments), C_3–5_ (short-chain intermediates), C_6–8_ (monomer derivatives), and C_9+_ (macromolecular fragments), reflecting structural stability hierarchies derived from PHXDT’s aromatic/cyclohexane moieties (six-membered rings) and monomeric building blocks (eight-carbon units).

C_1_ products (Figure 5b) displayed monotonic growth across all temperatures. High-temperature systems (≥3500 K) exhibited rapid equilibration followed by gradual decay, with peak values of 201 (3500 K) and 186 (4000 K), attributed to radical recombination mechanisms. C_2_ products (Figure 5c) demonstrated biphasic temperature-dependent behavior: continuous accumulation (final counts: 8–24) at 2000–2500 K versus transient peaks (53–79) followed by partial dissociation at ≥3000 K, indicating activation of secondary cracking reactions under extreme thermal conditions.

C_3–5_ intermediates (Figure 5d) showed sustained accumulation at moderate temperatures (2000–2500 K), dominated by depolymerization processes. At ≥3000 K, bimodal concentration profiles emerged (e.g., peaks at 82 and 98 for 4000 K at 5.425 ps and 11.85 ps, respectively), reflecting competitive chain scission and recombination pathways. The generation kinetics of C_6–8_ derivatives (Figure 5e) display pronounced temperature dependence. Peak emergence time demonstrates progressive acceleration from 35.4 ps at 2500 K to 4.025 ps at 4000 K, accompanied by a concurrent decrease in peak intensity from 61 to 51. This inverse correlation between thermal energy and temporal/spatial product distribution confirms that elevated temperatures facilitate direct dissociation of structural units while suppressing intermediate accumulation through enhanced reaction kinetics.

C_9+_ macromolecular fragments (Figure 5f) displayed triphasic evolution: rapid formation (backbone scission), slow decay (secondary cracking), and stabilization (carbonaceous residue formation). A pronounced thermal acceleration effect is evident in dissociation kinetics, where the 4000 K system achieves peak conversion efficiency of 74 at 0.85 ps, showing 15.7-fold rate enhancement compared to the 3000 K system (peak value of 52 at 13.4 ps). Final C_9+_ content stabilized near 10 across all systems, indicating structural convergence toward thermally stable carbon frameworks.

**Figure 6:**
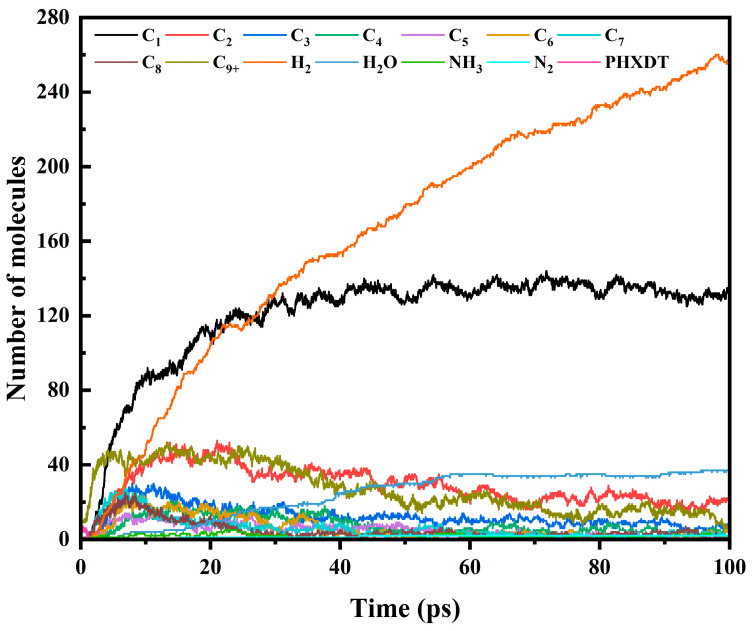
Evolution of different carbon-containing products and small molecule products containing hydrogen, nitrogen and oxygen at 3000 K.

Taking the pyrolysis evolution process of PHXDT under 3000 K conditions as the research object, this study delved into the distribution patterns of various carbon-containing products and small molecules such as hydrogen, nitrogen, and oxygen (as shown in Figure 6). During the initial pyrolysis stage (t < 0.05 ps), simultaneous structural decomposition of eight PHXDT molecular chains occurred, accompanied by rapid accumulation of C_9+_ macromolecular fragments. This intermediate reached maximum concentration at 0.075 ps followed by an exponential decay profile, ultimately stabilizing at approximately 10 molecules, indicative of limited carbonization/coking processes within the system. As depolymerization progressed (t > 0.4 ps), molecular weight distribution exhibited distinct C_1–8_ selective cleavage characteristics. Notably, hierarchical differentiation emerged in the formation kinetics of C_6–8_ components: Sequential initiation delays were observed with C_8_ (0.4 ps), C_7_ (0.425 ps), and C_6_ (1.525 ps), achieving maximum molecular counts of 25 (6.825 ps), 28 (6.725 ps), and 24 (7.4 ps), respectively. This kinetic hierarchy suggests aromaticity-driven secondary reactions preferentially stabilize seven-membered ring structures, potentially associated with conjugation energy differences in cyclic intermediates. Light products demonstrated rapid generation characteristics, with C_1–3_ molecules emerging earlier than C_4–5_ counterparts: C_1_ (1.175 ps), C_2_ (1.225 ps), and C_3_ (1.325 ps) preceded C_4_ (1.325 ps) and C_5_ (1.65 ps), revealing competitive mechanisms between backbone scission and radical recombination processes. Gas-phase analysis identified H_2_ as the dominant pyrolysis product (initial formation at 1.425 ps), ultimately constituting over 50% yield, with its high productivity correlating to C-H bond reorganization and dehydrogenation cyclization reactions. Heteroatom-containing species exhibited distinct behavior: NH_3_ (1.575 ps) and N_2_ (15.35 ps) showed significant kinetic retardation with final concentrations of merely 2 molecules, while H_2_O (4.85 ps) demonstrated progressive accumulation, reflecting higher thermal stability and stepwise elimination characteristics of oxygen-containing macromolecular structures.

### 3.3. Trends in Major Pyrolysis Products

**Figure 7:**
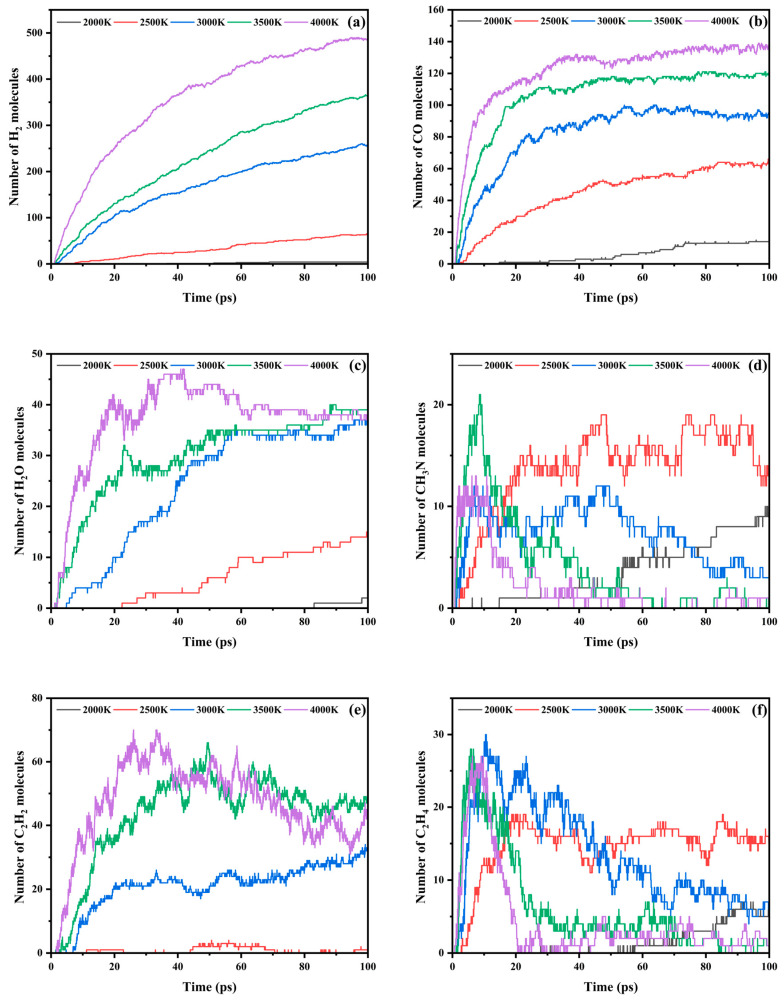
Evolution of (**a**) H_2_, (**b**) CO, (**c**) H_2_O, (**d**) CH_3_N, (**e**) C_2_H_2_, and (**f**) C_2_H_4_ during pyrolysis of PHXDT.

The formation kinetics of representative pyrolytic products (e.g., H_2_, CO, H_2_O, C_2_H_2_, C_2_H_4_ and CH_3_N) in c-SaPA decomposition were systematically investigated through ReaxFF-MD simulations. As demonstrated in Figure 7, all small-molecule products exhibited pronounced temperature-dependent formation characteristics across the thermal gradient (2000–4000 K). The statistical results of the number distribution of each product at 100 ps are detailed in Table 2. H_2_ evolution profiles (Figure 7a) revealed this primary product’s exponential growth pattern with temperature elevation. At 2000 K, merely 4 H_2_ molecules (0.5% of total hydrogen content) accumulated within 100 ps. Remarkably, the 4000 K system achieved a plateau phase after 90 ps with 484 H_2_ molecules, representing a 120-fold enhancement over the 2000 K yield. This accounted for 59.9% of total hydrogen atoms and 59.3% of molecular inventory, demonstrating pyrolysis behavior analogous to aramid materials.

The evolutionary behavior of oxygen-containing small molecules (Figure 7b,c) indicates that CO formation exhibits temperature-accelerated kinetics, with its initiation time decreasing by orders of magnitude as thermal energy increases. Systems maintained at temperatures equal to or exceeding 2500 K demonstrate a more than tenfold enhancement in CO generation kinetics compared to those at 2000 K. Notably, high-temperature regimes (3000–4000 K) manifested biphasic CO evolution: rapid production (0–20 ps, 100–130 molecules) followed by attenuated growth, culminating in CO accounting for >80% of total oxygen at 4000 K. Contrastingly, H_2_O displayed accumulation-depletion behavior at 4000 K, peaking at 47 molecules (40.925 ps) before gradual decay, while concurrent CO production persisted. Kinetic tracing has been demonstrated to reveal that the phenomenon originates from the dynamic redistribution process of elemental oxygen. That is to say, the O atoms in H_2_O are gradually transferred to CO, and the sum of the two and the total number of oxygen atoms in the system remain conserved at the late stage of pyrolysis.

The pyrolysis pathway analysis of carbon-containing products (Figure 7d–f) reveals distinct temperature-dependent behaviors. CH_3_N molecules exhibit monotonic accumulation in the low-temperature regime (2000–2500 K), while demonstrating dynamic generation-decomposition equilibrium at elevated temperatures (≥3000 K). The CH_3_N concentration peaks at 21 molecules (3500 K) and 13 molecules (4000 K) before gradually decaying to zero, indicating subsequent dehydrogenation reactions. C_2_H_2_ formation displays a notable temperature threshold effect, showing a parabolic evolution pattern above 3000 K with population peaking at 53 molecules at 26 ps (4000 K) before stabilizing at 25 molecules. C_2_H_4_ evolution exhibits characteristic secondary reaction behavior under high-temperature conditions (≥3000 K), rapidly reaching peak concentrations of 27–30 molecules within 6.325–10.675 ps followed by gradual depletion through dehydrogenation to C_2_H_2_. This depletion process demonstrates direct correlation with cyclohexane framework fragmentation kinetics.

### 3.4. Formation Pathways of Major Pyrolysis Products

To systematically elucidate the formation mechanisms of primary products during polyamide pyrolysis, this study employed a multicolor elemental labeling approach to visualize the dynamic evolution of molecular structures within the reaction system. The color scheme was defined as follows: black spheres represent carbon atoms; red corresponds to oxygen atoms; white denotes hydrogen atoms; dark blue identifies nitrogen atoms; and light blue specifically designates small-molecule pyrolytic products. Through this innovative characterization technique combined with product evolution analysis, we successfully deciphered the microscopic mechanisms underlying polyamide pyrolysis reactions.

**Figure 8:**
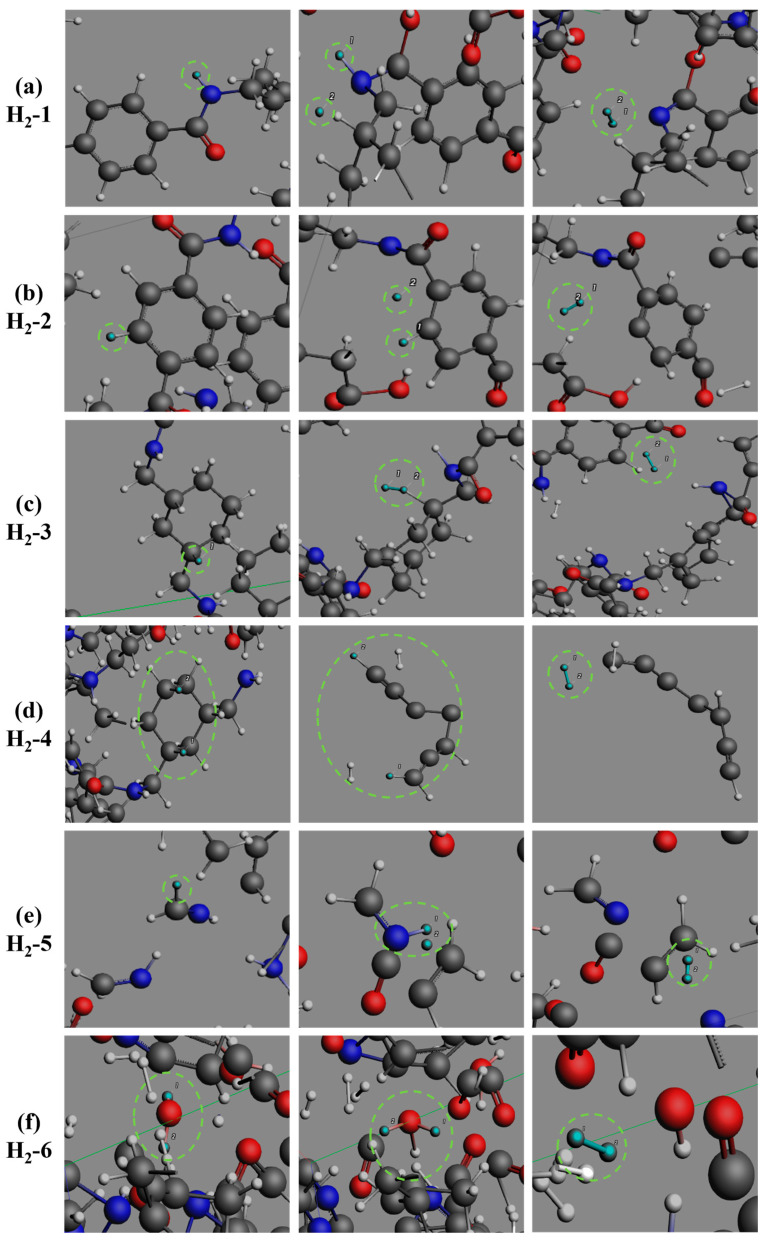
The formation process of H_2_ during the pyrolysis of PHXDT.

As detailed in the pathway analysis of Figure 8, H_2_ generation involves six critical reaction pathways and one foundational mechanism: (1) H· radical interaction with amide groups, (2) H· radical engagement with cyclohexane-structural hydrogens, (3) H· radical reaction with aromatic hydrogen atoms, (4) dehydrogenation of carbonaceous char, (5) concerted reaction between H· radicals and CH_3_N, (6) H· radical reaction with H_2_O, and (7) H· radical direct recombination, which persists as a fundamental mechanism throughout pyrolysis. The integrated study demonstrates that hydrogen formation primarily originates from the cleavage and recombination of C-H and N-H bonds. Kinetic analysis demonstrates that the rapid H_2_ generation during the initial to intermediate pyrolysis stages is predominantly driven by the synergistic effects of Pathways 1–2: the cleavage of N-H bonds in amide linkages and C-H bonds in cyclohexane structures not only induces polymer backbone depolymerization but also generates abundant H· radicals and reactive intermediates, establishing a positive feedback mechanism for radical chain reactions. The gradual H_2_ release in mid-to-late pyrolysis stages is attributed to secondary reactions dominated by Pathways 3–6, where C-H bond rupture in aromatic structures, further cracking of small molecules (e.g., CH_3_N, H_2_O), and C-H bond cleavage during carbon charring become primary contributors. Concurrently, Pathway 7 continuously scavenges residual H· radicals in the system. The synergistic interplay of these multi-stage reaction mechanisms correlates significantly with the H_2_ evolution kinetics depicted in Figure 7a.

**Figure 9:**
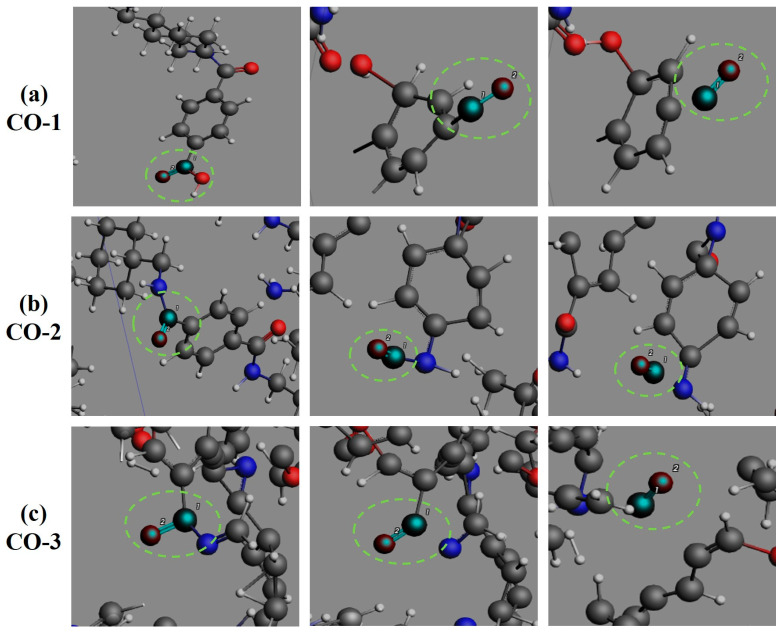
The formation process of CO during the pyrolysis of PHXDT.

**Figure 10:**
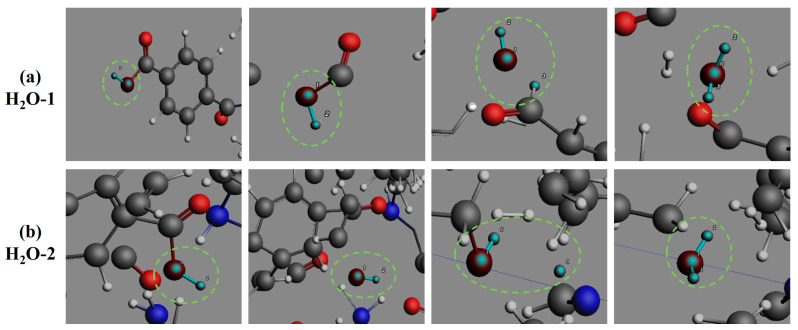
The formation process of H_2_O during the pyrolysis of PHXDT.

The oxygenated small-molecule product CO generation pathways (Figure 9) are governed by three principal routes: (1) decarboxylation of terminal carboxyl groups in PHXDT chains, where sequential cleavage of carbonyl C-O bonds generates OH· radicals followed by aromatic C-carbonyl bond rupture releasing CO; (2) intra-chain amide bond cleavage, where free amide groups formed through aromatic C-carbonyl bond dissociation undergo subsequent C-N bond scission; and (3) redox transformations of oxygenated intermediates (N-C-O and C-C-O structures derived from amide bond cleavage or H_2_O-carbon radical reactions). These pathways align with observed pyrolytic behavior, wherein early-stage CO release is dominated by decarboxylation while later stages exhibit contributions from radical-driven oxidation and secondary amide decomposition. This framework provides a molecular-level rationale for the dynamic CO production profile observed during PHXDT pyrolysis. For H_2_O production (Figure 10), two primary channels are identified: direct carboxyl group cleavage generating OH· radicals that subsequently combine with H·, and secondary dehydrogenation of alcohol intermediates (e.g., aliphatic hydroxyl compounds). Notably, OH· radicals serve as pivotal chain-transfer agents in the aqueous phase formation during PHXDT pyrolysis.

**Figure 11:**
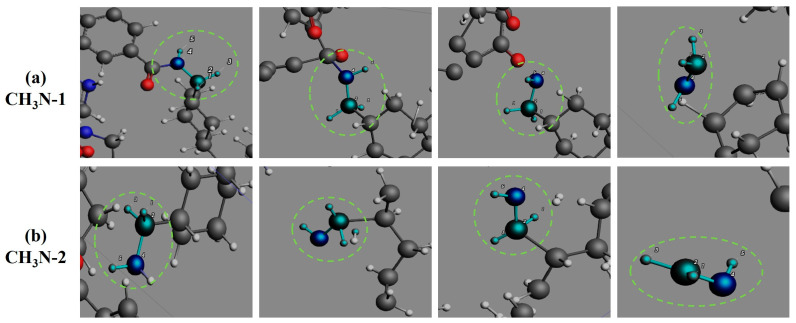
The formation process of CH_3_N during the pyrolysis of PHXDT.

**Figure 12:**
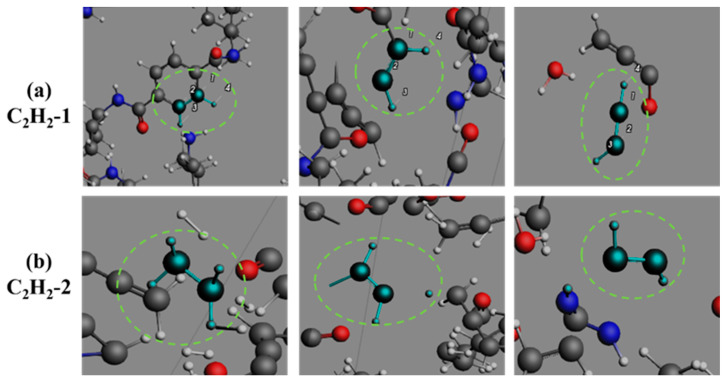
The formation process of C_2_H_2_ during the pyrolysis of PHXDT.

**Figure 13:**
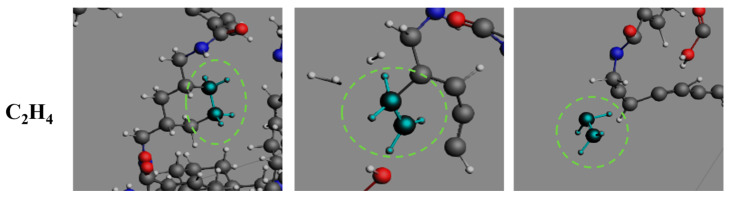
The formation process of C_2_H_4_ during the pyrolysis of PHXDT.

The formation of CH_3_N (Figure 11) proceeds through two distinct reaction sequences: one involving sequential cyclohexane chair-conformation distortion, amide C-N bond cleavage, and subsequent C-C bond rupture, and another via direct terminal amino group dissociation, collectively confirming amide groups as primary precursors. As shown in Figure 12, C_2_H_2_ formation occurs through two distinct routes. The primary pathway involves aromatic ring cleavage through two-stage benzene C-C bond rupture, predominantly occurring during initial pyrolysis stages. The secondary pathway emerges through C_2_H_4_ dehydrogenation in middle-late pyrolysis phases. These complementary routes comprehensively describe C_2_H_2_’s evolutionary mechanism, establishing aromatic rings and C_2_H_4_ as critical intermediates. Figure 13 illustrates the singular formation pathway for C_2_H_4_, generated via a two-step breaking of the C-C bond on cyclohexane during the initial PHXDT decomposition phase.

Collectively, the pyrolysis of PHXDT involves multi-pathway synergy governing product formation, with reaction mechanisms adhering to free radical chain processes mediated through cyclic radical relay. The cleavage dynamics exhibit pronounced bond dissociation energy hierarchy, where thermodynamic gradients drive systematic molecular skeleton deconstruction: C-C/C-H bond rupture in backbone structures precedes selective detachment of branched functional groups, ultimately achieving complete small-molecule liberation. Product distributions demonstrate explicit spatiotemporal correlation, with structural evolution initiating from ring-opening reorganization of cyclic conjugated systems, progressing through stepwise chain fragmentation, and culminating in highly condensed coke precursors. Critical to this pyrolysis network are the multi-modal transformations of carboxyl groups, amide linkages, and reactive intermediates (N-C-O/C-C-O/OH·/H·), which serve as kinetic hubs governing degradation pathways. Due to the absence of direct experimental data on PHXDT pyrolysis, indirect validation is required through comparison with structurally analogous systems (e.g., meta-aramid [41]). Future experimental characterization of PHXDT thermal decomposition (e.g., via TGA-MS or FT-IR) will aid in the establishment of quantitative benchmarks.

### 3.5. Role of Cyclohexane Structure in the Pyrolysis Process of PHXDT and Thermal Stability Design of c-SaPA Materials

Based on the findings presented in Section 3.1, Section 3.2, Section 3.3 and Section 3.4, the role of the cyclohexane structure in the pyrolysis reaction of PHXDT was elucidated, providing molecular-level guidance for the thermal stability design of c-SaPA materials.

The cyclohexane structure governs selective formation of small-molecule gaseous products in PHXDT pyrolysis. As a thermally labile structural unit, cyclohexane’s low C-C bond dissociation energy directly drives C_2–4_ hydrocarbon generation. During initial pyrolysis (<1 ps), chair conformation disruption initiates sequential C-C bond cleavage, preferentially yielding C_2_H_4_ (peaking at 30 molecules at 3000 K). In middle-late stages (>20 ps), C_2_H_4_ undergoes dehydrogenation to form C_2_H_2_ (peaking at 53 molecules at 4000 K).

Cyclohexane also modulates intermediate dynamics and carbonization pathways. Derived C_6–8_ intermediates (e.g., cyclohexane fragment recombination products) accumulate significantly at low temperatures (61 molecules at 2500 K), yet their high reactivity drives rapid decomposition at elevated temperatures (60% reduction by 100 ps, peak time shortened to 4 ps at 4000 K). H· radicals from cyclohexane C-H cleavage (contributing >30% total H_2_) promote dehydrogenation-driven carbonization, accelerating char precursor (C_9+_) formation (74 molecules peak at 0.85 ps, 4000 K). Ultimately, cyclohexane fragments transform into stable char through aromatization or condensation (C_9+_ stabilized at 10 molecules), demonstrating their dual role as transient intermediates and final products.

A comparative analysis was conducted on the synergistic effects between cyclohexane and benzene ring structures and their differential reactivity. Compared to the high stability of the benzene ring, cyclohexane undergoes pyrolysis more readily at lower temperatures due to its lower bond energy, with its conformational transformation identified as the key initiation step in the thermal decomposition process. Analysis of its molecular motion characteristics reveals that conformational freedom is a core influencing factor. Therefore, the introduction of sterically hindered groups to restrict the conformational freedom of cyclohexane can significantly increase the activation energy of the initial pyrolysis step, thereby delaying its thermal decomposition at the molecular motion level [21]. Benzene-dominated systems preferentially generate C_2_H_2_ products, while cyclohexane systems exhibit higher propensities for C_2_H_4_ and H_2_ formation, collectively determining product diversity. Notably, owing to its high dehydrogenation tendency (H_2_ yield accounting for 59.9%) and rapid carbon skeleton rearrangement capability, cyclohexane serves as a core structural unit for regulating the selectivity of small molecules and the rate of coking during PHXDT pyrolysis. Combined with the analysis of H_2_ formation pathways in Section 3.4, it can be established that the H· radical is the primary active species involved. Consequently, the introduction of H· radical scavengers (such as transition metal nanoparticles) can effectively modulate the dynamic migration behavior of active hydrogen, thereby precisely intervening in the free radical chain reactions during pyrolysis [56]. This strategy, by regulating the dynamic behavior of active hydrogen during pyrolysis, offers novel insights for enhancing the thermal stability of c-SaPA materials.

## 4. Conclusions

This investigation employed ReaxFF-MD simulations to develop a computational framework for the high-temperature pyrolysis process of PHXDT. Through systematic analysis of pyrolysis kinetics and the dynamic evolution of reaction pathways, we elucidated the formation mechanisms of primary pyrolytic products and uncovered the pivotal regulatory role of cyclohexane-based structural units in governing product distribution patterns. These findings not only establish a theoretical foundation for modulating pyrolytic product selectivity but also advance molecular design strategies informed by dynamic chemical behavior for developing high-performance c-SaPA material systems tailored for extreme-environment applications. These key findings can be summarized as follows:

(1) ReaxFF-MD simulations revealed distinct pyrolysis kinetics for multi-chain PHXDT systems, with calculated activation energy (107.55 kJ/mol) and a pre-exponential factor (9.64 × 10^13^ s^−1^) within the 2000–4000 K range. The temperature-dependent kinetics followed Arrhenius behavior, demonstrating accelerated backbone scission and radical-driven fragmentation at elevated temperatures, where decomposition times decreased exponentially from 18.2 ps (2000 K) to 0.625 ps (4000 K).
(2) Product distribution exhibited hierarchical fragmentation patterns: C_1–2_ products accumulated monotonically at low temperatures but stabilized rapidly under high thermal stress; C_3–5_ intermediates showed bimodal growth–decay behavior above 3000 K; C_6–8_ derivatives displayed accelerated peak emergence with reduced terminal quantities, indicating enhanced secondary cracking; C_9+_ macromolecular rapidly reached peak contents under elevated temperatures, followed by carbonization-driven stabilization at a diminished level. This progression confirmed a multi-stage mechanism: initial backbone rupture, intermediate macromolecular fragmentation, and terminal dehydrogenation and carbonization.
(3) The pyrolysis product distribution exhibits strong thermal field dependence. H_2_ dominates at elevated temperatures, showing exponential yield increase with temperature. CO formation exhibits rapid initial growth followed by stabilization. This process derives oxygen from structural oxygen-containing groups, engaging in dynamic competition with H_2_O for oxygen allocation. Thermal energy preferentially drives oxygen transfer toward CO formation. The characteristic peak phenomena observed in small-molecule hydrocarbons such as CH_3_N, C_2_H_2_, and C_2_H_4_ unveil critical transitions in rate-limiting steps governing formation and decomposition pathways during pyrolysis.
(4) The pyrolysis mechanism was governed by selective bond cleavage through radical chain reactions, generating dominant products (H_2_, CO, H_2_O, CH_3_N, C_2_H_2_, C_2_H_4_). Critical pathways included carboxyl group decarboxylation, amide bond dissociation, and transformations of N-C-O/C-C-O intermediates and OH·/H· radicals. These interconnected processes formed a reaction network where small-molecule release correlated with progressive carbon skeleton simplification, ultimately yielding thermally stable coke.
(5) The cyclohexane structure, in particular, has been identified as a key active structure that plays a crucial role in the regulation of the rapid generation of small molecule gases (H_2_, C_2_H_2_, C_2_H_4_, CH_3_N, etc.) and the acceleration of the conversion of intermediates to char. This dynamic process exhibits significantly higher reactivity in comparison to the benzene-ring structure, thereby providing a structurally sensitive basis for the directional modulation of the pyrolysis products of polyamides. The initial pyrolysis energy barrier of cyclohexane structure can be increased by restricting their conformational transition and the chain reaction can be inhibited by combining an H· radical trapping agent.

## Figures and Tables

**Table 1:** Number of molecules of products generated by pyrolysis at 100 ps at different temperatures.

	Total Fragments	C_1_	C_2_	C_3–5_	C_6–8_	C_9+_
2000 K	135	26	8	15	34	47
2500 K	327	103	24	35	32	46
3000 K	484	136	21	14	7	6
3500 K	690	158	31	15	15	13
4000 K	816	162	22	19	9	16

**Table 2:** Number of molecules of typical pyrolysis products at 100 ps at different temperatures.

	H_2_	CO	H_2_O	CH_3_N	C_2_H_2_	C_2_H_4_
2000 K	4	14	2	9	0	5
2500 K	65	62	15	13	1	16
3000 K	257	94	37	3	32	7
3500 K	364	121	39	0	35	1
4000 K	484	137	38	0	25	1

## Data Availability

The data that support the findings of this study are available upon reasonable request from the authors.

## References

[1] Endo T., Higashihara T. (2022). Direct Synthesis of Thermally Stable Semiaromatic Polyamides by Bulk Polymerization Using Aromatic Diamines and Aliphatic Dicarboxylic Acids. ACS Omega.

[2] García J.M., García F.C., Serna F., de la Peña J.L. (2010). High-Performance Aromatic Polyamides. Prog. Polym. Sci..

[3] Teng C., Li H., Liu J., Gu H., Kong H., Yu M. (2020). Effect of High Molecular Weight PPTA on Liquid Crystalline Phase and Spinning Process of Aramid Fibers. Polymers.

[4] Hernández G., Ferrero S., Reinecke H., Bartolomé C., Martinez-Ilarduya J.M., Álvarez C., Lozano Á.E. (2023). New Insights in the Synthesis of High-Molecular-Weight Aromatic Polyamides-Improved Synthesis of Rod-like PPTA. Int. J. Mol. Sci..

[5] Liu H., Xu M., Li X. (2024). Achievement of High-Reliability and High-Efficient Deposit of PA66 by Additive Friction Stir Deposition. Compos. Part B Eng..

[6] Mao L., Pan L., Ma B., He Y. (2022). Synthesis and Characterization of Bio-Based Amorphous Polyamide From Dimethyl Furan-2,5-Dicarboxylate. J. Polym. Environ..

[7] Yan G., Zhang G., Ren H., Li Y., Yang J. (2016). Synthesis and Characterization of Semiaromatic Polyamides with Dicyclohexane Units. RSC Adv..

[8] Zhang C. (2018). Progress in Semicrystalline Heat-Resistant Polyamides. e-Polymers.

[9] Lin X.B., Du S.L., Long J.W., Chen L., Wang Y.Z. (2016). A Novel Organophosphorus Hybrid with Excellent Thermal Stability: Core-Shell Structure, Hybridization Mechanism, and Application in Flame Retarding Semi-Aromatic Polyamide. ACS Appl. Mater. Interfaces.

[10] Zhang G., Yan G.M., Ren H.H., Li Y., Wang X.J., Yang J. (2015). Effects of a Trans- or Cis-Cyclohexane Unit on the Thermal and Rheological Properties of Semi-Aromatic Polyamides. Polym. Chem..

[11] Li J., Yi Y., Wang C., Lu W., Liao M., Jing X., Wang W. (2024). An Intrinsically Transparent Polyamide Film with Superior Toughness and Great Optical Performance. Polymers.

[12] Luo S., Dai J., Ji X., Chen J., Jiang Y., Li S., Mao T., Fan X. (2022). Synthesis of Novel Poly(Arylene Ether Amide) Containing Aliphatic Ring for Optical Property. High Perform. Polym..

[13] Zhong H., Deng J. (2021). Preparation and Chiral Applications of Optically Active Polyamides. Macromol. Rapid Commun..

[14] Li P.H., Wang C.Y., Li G., Jiang J.M. (2009). Highly Organosoluble and Transparent Polyamides Containing Cyclohexane and Trifluoromethyl Moieties: Synthesis and Characterization. Express Polym. Lett..

[15] Long J.W., Chen L., Liu B.W., Shi X.H., Lin X.B., Li Y.M., Wang Y.Z. (2020). Tuning the Pendent Groups of Semiaromatic Polyamides toward High Performance. Macromolecules.

[16] Arvanitoyannis I., Nikolaou E., Yamamoto N. (1994). Novel Biodegradable Copolyamides Based on Adipic Acid, Bis(p-Aminocyclohexyl)Methane and Several α-Amino Acids: Synthesis, Characterization and Study of Their Degradability for Food Packaging Applications: 4. Polymer.

[17] Cheng Y., Luo S., Wu Y., Huang T., Yu B., Zhu M., Yu H. (2024). One-Pot Synthesis of Copolyamide PA MXDT-MXD6 towards Oxygen-Barrier and High-Temperature Transparent Applications. Eur. Polym. J..

[18] Mutua F.N., Yang T., Gao Y., Zhu B., He Y. (2018). Preparation, Analysis, and Isothermal Crystallization Behavior of Poly[1,3-Bis(Aminomethyl)Cyclohexamethylene Oxamide]. J. Appl. Polym. Sci..

[19] Dong J., Fan Z. (2014). Nonisothermal Crystallization Behaviour of a Novel Cycloaliphatic Microcrystalline Poly(4,4′-Aminocyclohexyl Methylene Dodecanedicarboxylamide). Polym. Polym. Compos..

[20] Guo J., Li H., Xue B., Zhu D., Zhang X., Zhao H., Qiao X., Cui Z., Fu P., Zhao Q. (2021). A Synthesized Semi-Aromatic Copolyamaide through Synergy of Three Different Kinds of Monomers: Toward High Transparency, Excellent Heat Resistance and Melt Flowing Property. J. Appl. Polym. Sci..

[21] Li P.H., Wang C.Y., Li G., Jiang J.M. (2010). Synthesis and Characterization of Novel Polyamides Derived from 1,4-Bis((4-Amino-2-(Trifluoromethyl)Phenoxy)Methyl)Cyclohexane and Aromatic Dicarboxylic Acids. Polym. Bull..

[22] Murthy N.S., Bray R.G. (2003). Structure and Properties of Polyamide 6 and 4-Aminomethylcyclohexane Carboxylic Acid Copolymers with an Unusually Short Helical Pitch for Nylons. Polymer.

[23] Bradler P.R., Fischer J., Wallner G.M., Lang R.W. (2018). Characterization of Irradiation Crosslinked Polyamides for Solar Thermal Applications—Basic Thermo-Analytical and Mechanical Properties. Polymers.

[24] Bradler P.R., Fischer J., Wallner G.M., Lang R.W. (2019). Characterization of Irradiation Crosslinked Polyamides for Solar Thermal Applications-Fatigue Properties. Compos. Sci. Technol..

[25] Zhang Q., Zhu G.R., Xiao X.X., Liu Q.S., Jiang M., Guo D.M., Zhao H.B., Li W.D., Chen L., Liu B.W. (2023). Controllable Micro Cross-Linking towards Multifunctional Flame-Retardant Aliphatic Polyamide. Chem. Eng. J..

[26] Yang K., Liu Y., Zheng Z., Lu G., Tang Z., Chen X. (2022). Synthesis and Thermal Degradation Mechanism of a Semi-Aromatic Copolyamide from Renewable Sources. Polym. Degrad. Stab..

[27] Liu M., Li K., Yang S., Fu P., Wang Y., Zhao Q. (2011). Synthesis and Thermal Decomposition of Poly(Dodecamethylene Terephthalamide). J. Appl. Polym. Sci..

[28] Meng C., Liu X. (2022). Study on Thermal Degradation Kinetics of Bio-Based Semi-Aromatic High-Temperature Polyamide PA5T/56 and the Effect of Benzene Ring. Iran. Polym. J..

[29] Chen R., Zhang D., Xu X., Yuan Y. (2021). Pyrolysis Characteristics, Kinetics, Thermodynamics and Volatile Products of Waste Medical Surgical Mask Rope by Thermogravimetry and Online Thermogravimetry-Fourier Transform Infrared-Mass Spectrometry Analysis. Fuel.

[30] Zheng L., Wang M., Li Y., Xiong Y., Wu C. (2024). Recycling and Degradation of Polyamides. Molecules.

[31] Mao Q., Feng M., Jiang X.Z., Ren Y., Luo K.H., van Duin A.C.T. (2023). Classical and Reactive Molecular Dynamics: Principles and Applications in Combustion and Energy Systems. Prog. Energy Combust. Sci..

[32] Zhang F., Yang R., Lu D. (2023). Investigation of Polymer Aging Mechanisms Using Molecular Simulations: A Review. Polymers.

[33] Krishna S., Sreedhar I., Patel C.M. (2021). Molecular Dynamics Simulation of Polyamide-Based Materials-A Review. Comput. Mater. Sci..

[34] van Duin A.C.T., Dasgupta S., Lorant F., Goddard W.A. (2001). ReaxFF:  A Reactive Force Field for Hydrocarbons. J. Phys. Chem. A.

[35] AlAreeqi S., Bahamon D., Polychronopoulou K., Vega L.F. (2022). Insights into the Thermal Stability and Conversion of Carbon-Based Materials by Using ReaxFF Reactive Force Field: Recent Advances and Future Directions. Carbon.

[36] Zheng Y.B., Zhang Q., Mei S.N., Wang W.Q., Shi J., Yu Q.W., Zhai G.H., Yang J.M. (2023). Molecular Dynamic Simulation of LiH–H_2_O Reactions Using the ReaxFF Reactive Force Field. Int. J. Hydrogen Energy.

[37] Kowalik M., Ashraf C., Damirchi B., Akbarian D., Rajabpour S., van Duin A.C.T. (2019). Atomistic Scale Analysis of the Carbonization Process for C/H/O/N-Based Polymers with the ReaxFF Reactive Force Field. J. Phys. Chem. B.

[38] Senftle T.P., Hong S., Islam M.M., Kylasa S.B., Zheng Y., Shin Y.K., Junkermeier C., Engel-Herbert R., Janik M.J., Aktulga H.M. (2016). The ReaxFF Reactive Force-Field: Development, Applications and Future Directions. Npj Comput. Mater..

[39] Yilmaz D.E., Woodward W.H., van Duin A.C.T. (2021). Machine Learning-Assisted Hybrid ReaxFF Simulations. J. Chem. Theory Comput..

[40] Yang J., Zheng Y., Shi J., Jia Y., Li J., Zhang Q., Wang W., Yu Q. (2023). Molecular Dynamic Simulation of Ni–Al Alloy–H2O Reactions Using the ReaxFF Reactive Force Field. ACS Omega.

[41] Yin F., Tang C., Wang Q., Liu X., Tang Y. (2018). Molecular Dynamics Simulations on the Thermal Decomposition of Meta-Aramid Fibers. Polymers.

[42] Guo G., Fan K., Guo Z., Guo W. (2023). Pyrolysis Behavior of Automotive Polypropylene Plastics: ReaxFF Molecular Dynamics Study on the Co-Pyrolysis of Polypropylene and EPDM/POE. Energy.

[43] Li X., Han Y., Qu J., Chen Q., Wei Y., Hou G., Liu J. (2023). ReaxFF Molecular Dynamics Simulation of the Thermal Decomposition Reaction of Bio-Based Polyester Materials. Phys. Chem. Chem. Phys..

[44] Zhou S., Zhang L., Zou L., Ayubi B.I., Wang Y. (2024). Mechanism Analysis and Potential Applications of Atomic Oxygen Erosion Protection for Kapton-Type Polyimide Based on Molecular Dynamics Simulations. Polymers.

[45] Chen T.B.Y., Yuen A.C.Y., Lin B., Liu L., Lo A.L.P., Chan Q.N., Zhang J., Cheung S.C.P., Yeoh G.H. (2021). Characterisation of Pyrolysis Kinetics and Detailed Gas Species Formations of Engineering Polymers via Reactive Molecular Dynamics (ReaxFF). J. Anal. Appl. Pyrolysis.

[46] Zhao T., Li T., Xin Z., Zou L., Zhang L. (2018). A ReaxFF-Based Molecular Dynamics Simulation of the Pyrolysis Mechanism for Polycarbonate. Energy Fuels.

[47] Deepa P., Sona C., Jayakannan M. (2006). Synthesis and Investigation of the Effect of Nematic Phases on the Glass-Transition Behavior of Novel Cycloaliphatic Liquid-Crystalline Poly(Ester Amide)s. J. Polym. Sci. Part Polym. Chem..

[48] Guo W., Fan K., Guo G., Wang J. (2022). Atomic-Scale Insight into Thermal Decomposition Behavior of Polypropylene: A ReaxFF Method. Polym. Degrad. Stab..

[49] Martínez L., Andrade R., Birgin E.G., Martínez J.M. (2009). PACKMOL: A package for building initial configurations for molecular dynamics simulations. J. Comput. Chem..

[50] Kato T., Yamada Y., Nishikawa Y., Ishikawa H., Sato S. (2021). Carbonization Mechanisms of Polyimide: Methodology to Analyze Carbon Materials with Nitrogen, Oxygen, Pentagons, and Heptagons. Carbon.

[51] Chen J., Meng T., Wang Q., Bai Y., Jiaqiang E., Leng E., Zhang F., Liao G. (2022). Study on the Mechanisms of Epoxy Resin Gasification in Supercritical Water by Molecular Dynamics and Experimental Methods. Chem. Eng. J..

[52] Tan S., Do D.D., Nicholson D. (2020). Consistency of NVT, NPT, µVT and Gibbs (NV^2^T and NPT) with Kinetic Monte Carlo Schemes. Chem. Eng. J..

[53] Bosko J.T., Todd B.D., Sadus R.J. (2005). Molecular Simulation of Dendrimers and Their Mixtures under Shear: Comparison of Isothermal-Isobaric (NpT) and Isothermal-Isochoric (NVT) Ensemble Systems. J. Chem. Phys..

[54] Xu M., Di J., Wu Y., Meng X., Ji H., Jiang H., Li J., Lu Q. (2023). Insights into the Pyrolysis Mechanisms of Epoxy Resin Polymers Based on the Combination of Experiments and ReaxFF-MD Simulation. Chem. Eng. J..

[55] Zhang F., Cao Y., Liu X., Xu H., Lu D., Yang R. (2021). How Small Molecules Affect the Thermo-Oxidative Aging Mechanism of Polypropylene: A Reactive Molecular Dynamics Study. Polymers.

[56] Sai T., Ran S., Guo Z., Song P., Fang Z. (2022). Recent Advances in Fire-Retardant Carbon-Based Polymeric Nanocomposites through Fighting Free Radicals. SusMat.

